# Synergistic enhancement in the microelectronic properties of poly-(dioctylfluorene) based Schottky devices by CdSe quantum dots

**DOI:** 10.1038/s41598-020-61602-1

**Published:** 2020-03-16

**Authors:** Fida Muhammad, Muhammad Tahir, Muhammad Zeb, Muttanagoud N. Kalasad, Suhana Mohd Said, Mahidur R. Sarker, Mohd Faizul Mohd Sabri, Sawal Hamid Md Ali

**Affiliations:** 1 Department of Physics, Faculty of Physical and Numerical Sciences, Abdul Wali Khan University Mardan, Mardan, 23200 Khyber Pakhtunkhwa Pakistan; 2 Electronics & Nanoscale Engineering, School of Engineering, University of Glasgow, Glasgow, G12 8QQ UK; 3 Department of Electrical Engineering, Faculty of Engineering, University of Malaya, Kuala Lumpur, 50603 Malaysia; 4 Department of Physics, Davangere University, Davangere, 577 007, Karnataka India; 5 Department of Mechanical Engineering, Faculty of Engineering, University of Malaya, Kuala Lumpur, 50603 Malaysia; 6 Department of Electric, Electronics and System Engineering, Faculty of Engineering and Built Environment, Universiti Kebangsaan Malaysia, Bangi, 43600 Malaysia

**Keywords:** Quantum dots, Electronic devices, Organic-inorganic nanostructures, Polymers, Quantum dots

## Abstract

This paper reports the potential application of cadmium selenide (CdSe) quantum dots (QDs) in improving the microelectronic characteristics of Schottky barrier diode (SBD) prepared from a semiconducting material poly-(9,9-dioctylfluorene) (F8). Two SBDs, Ag/F8/P3HT/ITO and Ag/F8-CdSe QDs/P3HT/ITO, are fabricated by spin coating a 10 wt% solution of F8 in chloroform and 10:1 wt% solution of F8:CdSe QDs, respectively, on a pre-deposited poly(3-hexylthiophene) (P3HT) on indium tin oxide (ITO) substrate. To study the electronic properties of the fabricated devices, current-voltage (*I–V*) measurements are carried out at 25 °C in dark conditions. The *I–V* curves of Ag/F8/P3HT/ITO and Ag/F8-CdSe QDs/P3HT/ITO SBDs demonstrate asymmetrical behavior with forward bias current rectification ratio (RR) of 7.42 ± 0.02 and 142 ± 0.02, respectively, at ± 3.5 V which confirm the formation of depletion region. Other key parameters which govern microelectronic properties of the fabricated devices such as charge carrier mobility (µ), barrier height (ϕ_b_), series resistance (R_s_) and quality factor (n) are extracted from their corresponding *I–V* characteristics. Norde’s and Cheung functions are also applied to characterize the devices to study consistency in various parameters. Significant improvement is found in the values of R_s_, n, and RR by 3, 1.7, and 19 times, respectively, for Ag/F8-CdSe QDs/P3HT/ITO SBD as compared to Ag/F8/P3HT/ITO. This enhancement is due to the incorporation of CdSe QDs having 3-dimensional quantum confinement and large surface-to-volume area. Poole-Frenkle and Richardson-Schottky conduction mechanisms are also discussed for both of the devices. Morphology, optical bandgap (1.88 ± 0.5 eV) and photoluminescence (PL) spectrum of CdSe QDs with a peak intensity at 556 nm are also reported and discussed.

## Introduction

Junction between different materials has a vital role in electronic and optoelectronic devices and is, therefore, one of the significant parts that govern the performance of a device. Well-controlled and easily tunable interfacial properties are always desirable in many electronic and optoelectronic devices. Among different types of junctions in devices, metal–semiconductor (MS) structures provide their role in the form of Schottky barrier diodes (SBDs)^1^. SBDs play a central role in the device functioning^2^ as well as is the most widely used type of junctions in semiconductor devices^3^, solar cells^4^ and field effect transistors (FETs)^5^. Mostly, commercially available electronic devices are based on inorganic semiconductors due to their high stability and performance^6^. However, due to high cost, high temperature complex processability and mechanical brittleness, inorganic semiconductors are not favorable. On the other hand, organic semiconducting materials are rich in pi-conjugated structures and have gained remarkable attention due to overcoming the issues with inorganic semiconductors. Organic semiconductors offer low temperature processability, low cost, mechanical flexibility, simple methods for device fabrication such as spin coating, drop casting, spray coating, inkjet-printing etc.^7,8^. Particularly, polymeric semiconductors are of great interest due to their solution processability in many organic solvents and/or in water. This property also allows one to tune the properties of polymer as desirable by making blend/suspension with other functional materials and nanoparticles^9–11^. Blending nanoparticles into polymer matrix can lead to wonderful electronic and optoelectronic properties by virtue of high surface-to-volume ratio of the nanomaterials.

Among plenty of conjugated polymers, polyfluorene is a class of semiconducting materials owing to their efficient emission, relatively high charge mobility and good thermal, mechanical and chemical stabilities^12^. A diversity of intrinsic properties of polyfluorene and its derivatives escort to the progress of high molecular weight material with easy synthesis and purification^13,14^. Polyfluorenes have demonstrated good performance in organic light emitting diodes (OLEDs)^15^, organic photovoltaic (OPV) devices^16^ and thin film FETs^17^. Poly-(9,9-dioctylfluorene) (F8) is semiconducting polymer from the polyfluorene family that has high hole mobility along with interesting electronic and optoelectronic properties^18^. F8 is soluble in many organic solvents like toluene, chloroform, p-xylene etc^19^. and can be easily deposited by spin coating, dip coating and drop casting techniques. One of the most important advantages of F8 solution is that it allows making blends for tuning its properties by mixing or combining two or more functional nanomaterials or polymers to attain the required characteristics without any chemical reactions^13^. Polymer-nanomaterials blends lead to the production of such nanocomposites which possess novel electronic, optical and structural properties which are special from their individual constituents^20,21^. Recently, inorganic nanoparticles and nanocomposites have been successfully used in variety of polymers to improve the efficiency of various devices like solar cells^22^, thermoelectric generators^23^ and sensors^24^. Hence, polymer-inorganic nanocomposites jointly demonstrate fascinating characteristics because of superior electrical properties of inorganic nanomaterials along with mechanically flexible as well as simply processable polymer matrix. In our previous work^25^, a blend of F8 with CdSe QDs –F8-CdSe QDs– has been successfully employed in the fabrication of humidity and temperature sensors demonstrating outstanding results.

CdSe is a well-known semiconducting material that acquires wonderful optoelectronic properties^26–28^ as revealed by its potential applications in energy harvesting, optical amplifiers, photodiodes and lasers^29–31^. At present times, nanomaterials have got significant attention in many fields because of their interesting properties different from the bulk and a lot of manufacturing uses. Nanomaterials can be found in different shapes like 3-dimensional nanospheres, 2-dimensional thin films or nanosheets, 1-dimensional nanotubes or nanowires and 0-dimensional materials known as QDs. Prospective applications of many semiconductor QDs have been investigated for Photovoltaics, FETs, biomedical and lasers. Whereas, CdSe QDs are the most interesting materials to researchers because of large surface-to-volume ratio, three-dimentional (3D) quantum confinement and highly stable at room as well as moderately elevated temperatures. Usually, CdSe QDs are found in a variety of structures such as zinc blende cubic (sphalerite), wurtzite (hexagonal) and rock-salt (cubic). Nevertheless, a lot of studies have been done to investigate CdSe QDs alone for the electronic and optoelectronic applications. Though, there is a lot of space to investigate and understand its role together within a polymer matrix for improving electronic, mechanical and thermal properties as well as stability of polymer matrix. Hence, CdSe QDs have the potential to be used in variety of devices’ applications.

Herein, we study the effect of CdSe QDs in enhancing the microelectronic properties of SBD prepared from F8. By spin coating F8-CdSe QD nanocomposite solution, Ag/F8-CdSe QDs/P3HT/ITO SBD is fabricated. Electronic properties of the SBD are significantly improved as compared to that of Ag/F8/P3HT/ITO device. The values of different electronic parameters of the fabricated devices have been extracted from the conventional *I–V* characterization method, Cheung’s functions and Norde’s technique to validate the consistency among the values obtained. Thin film morphology, PL spectrum and charge conduction mechanism through F8-CdSe nanocomposite device have also been studied.

## Experimental Work

### Device fabrication

**Figure 1:**
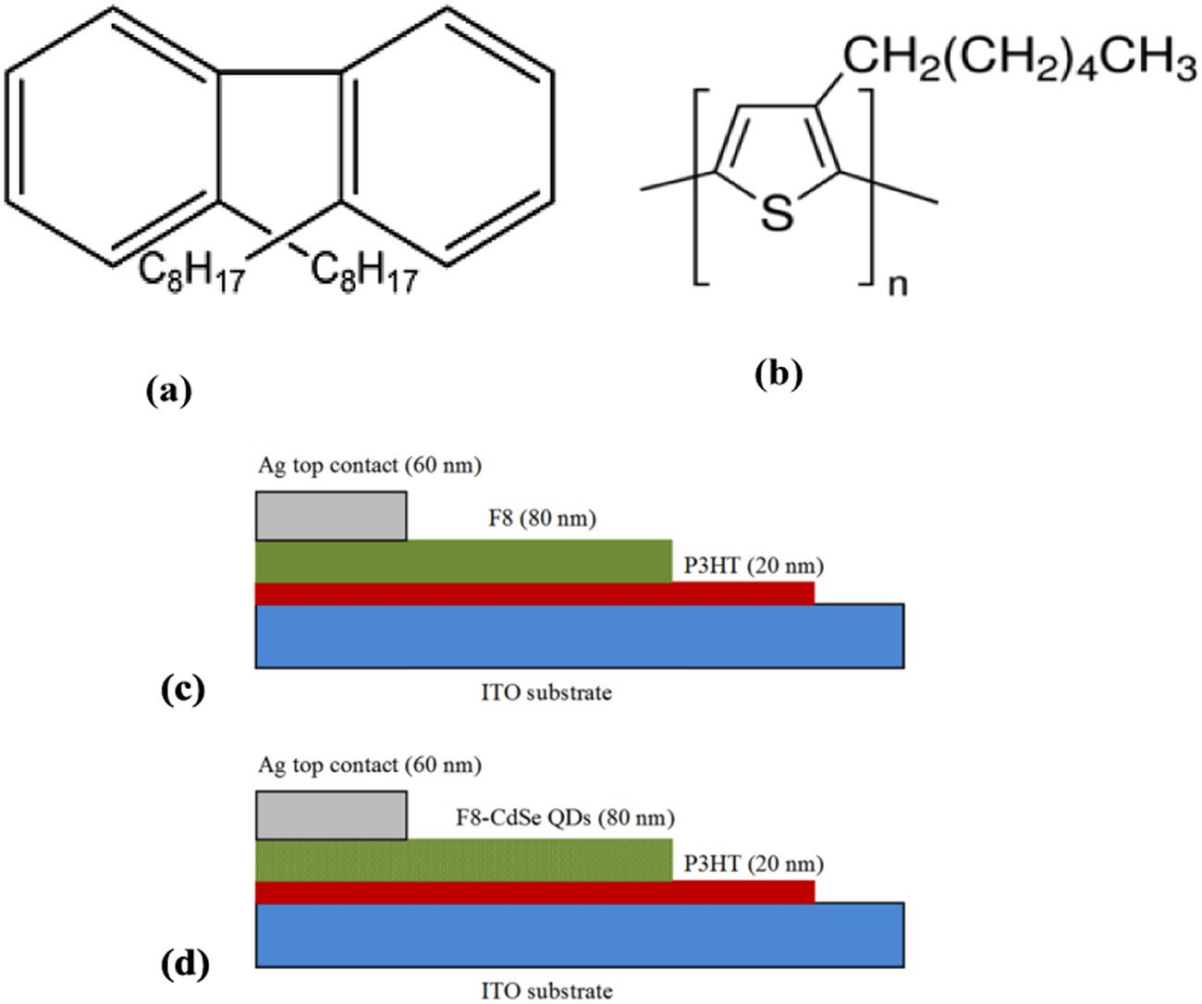
(**a**) molecular structure of F8, (**b**) molecular structure of P3HT, (**c**) **c**ross-sectional view of Ag/F8/P3HT/ITO SBD (**d**) **c**ross-sectional view of Ag/F8-CdSe QDs/P3HT/ITO.

The F8 polymer was obtained from Cambridge Display Technology (CDT), UK. CdSe quantum dots were synthesized by the method reported in our previous work^25^. The commercially available P3HT was purchased from Sigma Aldrich. Figure 1(a,b) show the molecular structures of F8 and P3HT. A 10 wt.% uniform solution of F8 and 1 wt.% suspension of CdSe QDs in chloroform were prepared and then were blended together by to make a uniform suspension of F8-CdSe QDs nanocomposite. The ITO-coated glass was used as a substrate for SBDs that was cleaned in acetone and isopropanol by using ultrasonic bath for 10 min and then dried under the continuous flow of dry nitrogen gas. A buffer layer of P3HT of thickness 20 nm was deposited on the cleaned ITO coated glass substrate by using spin coater at the rate 2000 rpm for 20 s. The P3HT layer was annealed for two hours by using hot plat at 50 °C in nitrogen environment to evaporate moisture from the layer. After the buffer layer, an 80 nm thin film of F8-CdSe blend was deposited using the spin coating technique on the pre-coated P3HT layer over a glass substrate coated with ITO. The vacuum thermal evaporation was used to deposit a 60 nm thick Ag electrode on F8-CdSe active layer to complete the fabrication of Ag/F8-CdSe QDs/P3HT/ITO SBD. Similar procedure is followed for the development of Ag/F8/P3HT/ITO SBD where no CdSe QDs are used. Figure 1(c,d) show the schematic structures of the fabricated Ag/F8/P3HT/ITO and Ag/F8-CdSe QDs/P3HT/ITO SBDs, respectively.

### Device characterization

Keithley-228A and Keithley-196 system DMM voltage-current source was used to measure various electrical characteristics. SEM measurements are carried out using JEOL JSM-840 having electron gun with operating voltage 0.2 to 40 kV. UV-Vis and PL spectra were measured by using Hewlett Packard 8453 and Ocean Fiber Optic Spectrometer USB4000.

## Results and Discussion

### Material characterization

**Figure 2:**
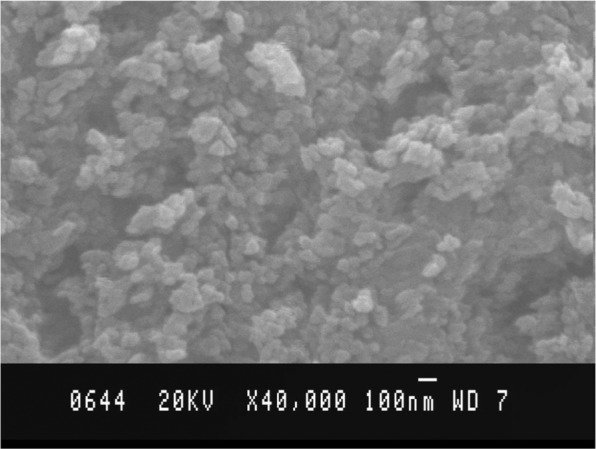
SEM image of CdSe QDs.

Figure 2 shows SEM image of CdSe film on glass substrate. This figure shows roughness in the film which may possibly be caused by irregular grains orientation at the time of deposition of the film. The figure also illustrates that distribution of the grains is fairly uniform, however, orientations are random. The roughness of film is believed to be responsible for the trapping of charge carriers in the device and thus leading it towards non ideal response and lowers the device mobility. The trapped charge carriers would thus require more energy for its mobilization and thermionic emission becomes dominant in the junction.

**Figure 3:**
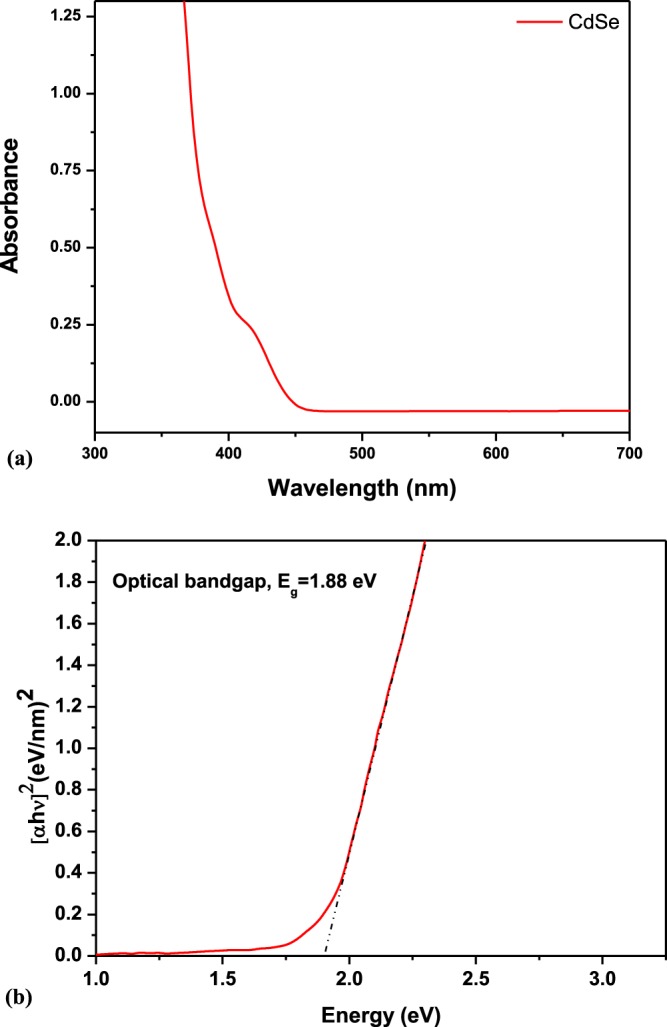
(**a**) UV-vis absorption spectrum of CdSe QDs (**b**) Tauc’s plot for bandgap measurement of CdSe QDs.

Figure 3(a) shows the UV-visible spectrum of CdSe QDs. The spectrum shows a broad absorption with strong intensities around violet and blue regions; which gradually decays in the visible range with increase in wavelength. To find the optical bandgap of CdSe QDs, the Tauc’s equations –as given by (1) –are applied to Fig. 3(a) that gives Fig. 3(b). In Tauc’s eaquation, the absorption coefficient “α” of the thin film is related to the incoming photon energy (hν) as follows:

1 $$\alpha h\nu =C{(h\nu -{E}_{g})}^{\vartheta }$$

where h is Planck’s constant, ν is the photon’s frequency, C is a proportionality constant and also known as the band tailing parameter, E_g_ is optical bandgap and $\vartheta$ is the power factor of the transition mode. The value of the exponent ϑ depends on the nature of material, whether it is crystalline, polycrystalline or amorphous which in turn give information about the electronic transition i.e., allowed or forbidden and direct or indirect transition. These transitions are listed in Table 1.

**Table 1:** Various optical transitions corresponding to their respective power factors.

Transition Mode	Status	Power Factor ($\vartheta$)
Direct	Allowed	½
	Forbidden	3/2
Indirect	Allowed	2
	Forbidden	3

Normally, they are only the allowed transitions which are dominant in the main absorption processes, giving either ϑ = 1/2 or  ϑ= 2, for direct and indirect transitions, respectively. However, in this case, for CdSe ϑ= ½, so E_g_ in Eq. (1) is direct allowed bandgap.

**Figure 4:**
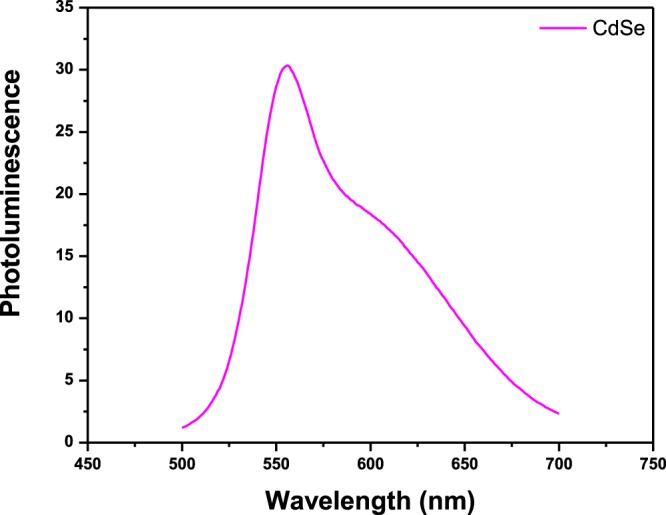
Photoluminescence spectrum of CdSe QDs at room temperature.

The measured optical bandgap, E_g_, for CdSe QDs having ~5 nm diameter is 1.88 ± 0.05 eV which is in good agreement with the values reported elsewhere^31,32^. Whereas, the bandgap of bulk CdSe is reported to be ~1.74 eV. However, the observed blue-shift in the optical band is ~ 0.14 eV. Figure 4 shows a broad photoluminescence (PL) spectrum of CdSe QDs. The dominant band in the spectrum is at ~ 556 nm^33^. Such a broad range of the PL spectrum implies to the presence of different size distribution of CdSe QDs. Here, this transition is direct band-to-band transition in CdSe QDs.

### Device characterization

**Figure 5:**
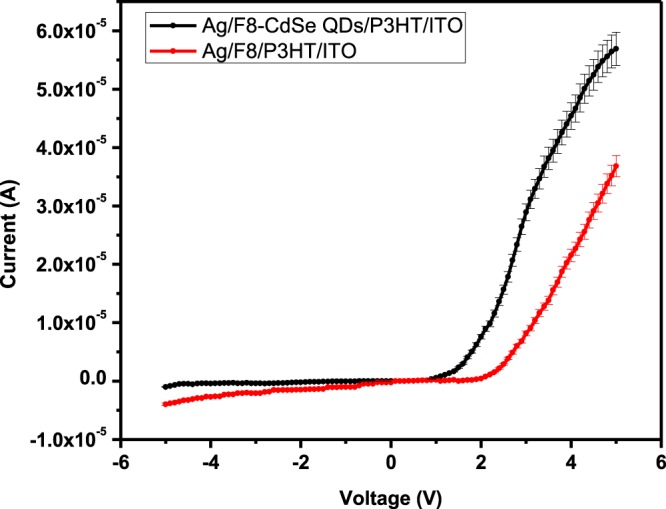
Current-voltage (*I–V*) characteristics of Ag/F8-CdSe/P3HT/ITO and Ag/F8/P3HT/ITO SBDs at 300 K.

The *I–V* characteristics of the Ag/F8-CdSe/P3HT/ITO Schottky diode have been measured to extract the junction parameters, such as rectification ratio ‘*RR*’, ideality factor ‘*n*’, barrier height ‘*ϕ*_*b*_’, reverse saturation current ‘*I*_0_’, and series resistance ‘*Rs*’ which participate a significant role in device performance. The behavior of *I–V* curves have also been examined to describe the properties that govern transport mechanism of the device. The nonlinear *I–V* characteristics, obtained at room temperature, of Ag/F8/P3HT/ITO and Ag/F8-CdSe/P3HT/ITO Schottky diode are given in Fig. 5 showing asymmetric behavior. The graph shows a clear enhancement in the junction behavior for CdSe blended F8 film. The forward bias *I –V* slope depends on ‘*n*’ and *I*_0_. In the device, recombination processes’ information may be obtained from *n* and thus may be compared with an ideal device^5^. However, in the reverse bias *I*_0_ shows the possibility of crossing the barrier by charge carriers^34,35^. The *RR* was measured to be 7.42 ± 0.02 and 142 ± 0.02 at ±3.7 V and the turn on voltage is measured to be 3.4 V and 1.7 V for Ag/F8/P3HT/ITO and Ag/F8-CdSe/P3HT/ITO, devices, respectively. The enhancement in these parameters is attributed to CdSe quantum dots which are believed to conduct more charges than F8 due to high surface to volume ratio of quantum dots.

The analysis based on Fig. 5 leads to the point that thermionic emission is the dominant mechanism in the junction, according to which,

2 $$I={I}_{0}\left[\exp \left(\frac{q(V-I{R}_{s})}{nkT}\right)-1\right]$$

The saturation current I_0_ is given as:

3 $${I}_{0}=A{A}^{\ast }{T}^{2}\exp \left(\frac{-q{\phi }_{b}}{kT}\right)$$

here q is electronic charge, V is voltage applied, A* is the effective Richardson constant equal to 1.3 × 10^5^ A/cm^2^ K^2^ for ITO, A is the effective diode area (0.09 cm^2^), T is the absolute temperature, k = 1.38 × 10^−23^ J/K is the Boltzmann constant. The following relation can be used to determine the value of *n*:

4 $$n=\frac{q}{kT}\frac{dV}{d(\mathrm{ln}\,I)}$$

**Figure 6:**
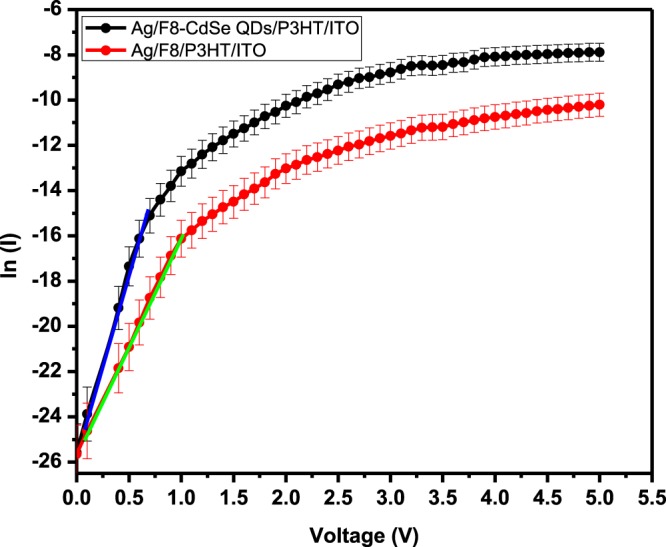
Semi-logarithmic (*I–V*) characteristics of Ag/F8-CdSe/P3HT/ITO and Ag/F8/P3HT/ITO SBDs at 300 K.

From ln *I*–*V* graph of Fig. 6, *I*_0_ is determined to be 6.2 × 10^−10^ A and 2.39 × 10^−11^ A for Ag/F8/P3HT/ITO and Ag/F8-CdSe/P3HT/ITO, respectively, by extrapolating it to zero bias. The junction barrier height can be computed from the relation;

5 $${\phi }_{b}=\frac{kT}{q}\,\mathrm{ln}\left(\frac{A{A}^{\ast }{T}^{2}}{{I}_{o}}\right)$$

The junction’s ideality/quality factor (*n*) shows the comparison of fabricated junction with ideal one and is found using Eq. (4) and the slope of the linear region of Fig. 6. Usually, *n* is observed to be greater than 1^36^ and this higher value is attributed to oxide layer on substrate surface, series resistance or interfacial states^37^. In our case, the value of *n* is calculated to be 4.03 and 2.38 for Ag/F8/P3HT/ITO and Ag/F8-CdSe/P3HT/ITO device respectively. The values of *n* greater than unity possibly be attributed to the trapping of charge carriers in randomly oriented and non-uniform grains in the film as was observed in the SEM image of Fig. 2. The barrier height is calculated to be 1.09 ± 0.05 and 1.17 ± 0.05 eV for Ag/F8/P3HT/ITO and Ag/F8-CdSe/P3HT/ITO device respectively using Eq. (5).

**Figure 7:**
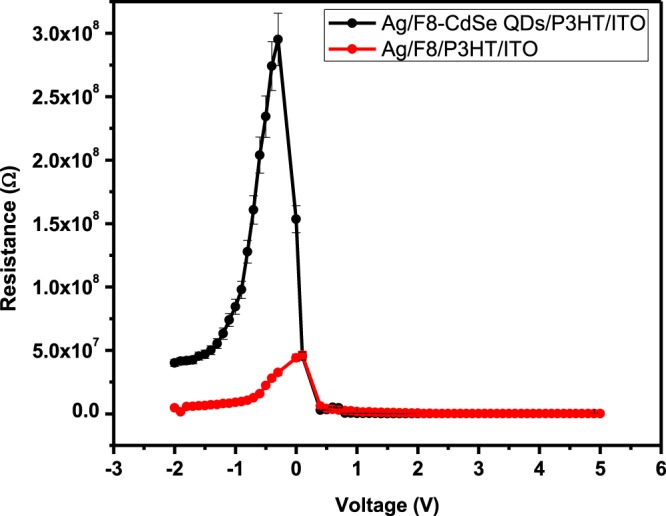
Interfacial resistance-voltage (*R*_*j*_*-V*) curves of Ag/F8-CdSe/P3HT/ITO and Ag/F8/P3HT/ITO SBDs at 300 K.

A graph of external applied voltage versus junction resistance was plotted to compute the diode’s series and shunt resistance, where $\,R=dV/dI$. Figure 7 demonstrate the graph between junction resistance and applied voltage for the junction diodes Ag/F8/P3HT/ITO and Ag/F8-CdSe/P3HT/ITO. When the forward bias voltage is high the value of junction resistance is minimum which demonstrate series resistance *R*_*s*_ and is found to be 269 ± 0.10 and 78.2 ± 0.10 kΩ, respectively, for Ag/F8/P3HT/ITO and Ag/F8-CdSe/P3HT/ITO Schottky junction diode whereas the shunt resistance *R*_*sh*_ is the maximum resistance in the reverse bias having a value of 46.2 ± 0.10 and 295 ± 0.10 MΩ for Ag/F8/P3HT/ITO and Ag/F8-CdSe/P3HT/ITO junction diode, respectively. The series resistance is a parasitic component in the junction which deviate the diode behavior from ideal to non-ideal. Table 2 presents the overall comparison of different parameters for both of the devices.

**Table 2:** Comparison of various key parameters of the fabricated SBDs.

Device	(*RR*)	*I*_0_ (A)	*n*	*Φ*_*B*_ (eV)	*R*_*s*_ (kΩ)	*R*_*sh*_ (MΩ)
Ag/F8/P3HT/ITO	7.42	6.23 × 10^−10^	4.03	1.09	269	46.2
Ag/F8-CdSe QDs/P3HT/ITO	142	2.39 × 10^−11^	2.38	1.17	78.2	295

**Figure 8:**
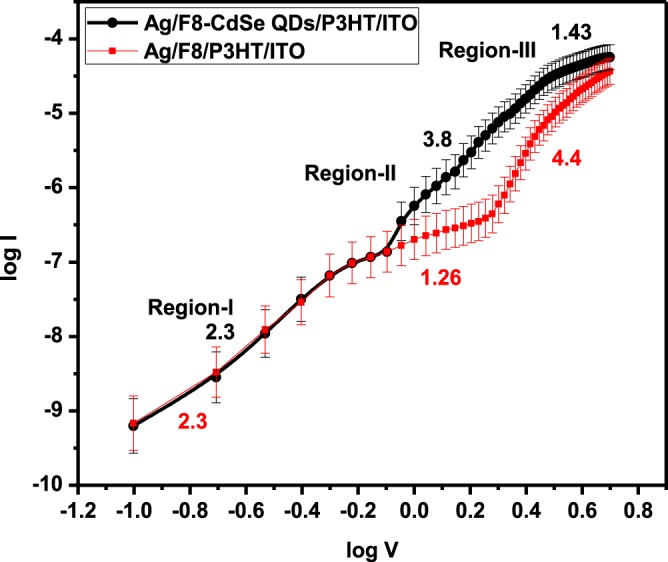
Double-logarithmic curves of *I–V* curve of Ag/F8-CdSe/P3HT/ITO and Ag/F8/P3HT/ITO SBDs.

Figure 8 shows the log(*I)* vs. log(*V)* curves to study the forward bias transport mechanism in both of the devices, with and without CdSe QDs, which illustrate the power law performance of current, i.e., I ∝ V^m^ ^38^ having different value of *m* (slope of the region) for different positions. The ohmic region is dominant for ${\rm{m}}\approx 1$ where current is directly proportional to the applied voltage, space charge limited current (SCLC) for ${\rm{m}}\approx 2$ where current and voltage are related by a quadratic relationship. The existence of SCLC regime can be observed in two cases; (i) when the traps present at the junction are either partially filled i.e., the trapping parameter: θ < 1 or fully filled θ = 1. Furthermore, the trapped charge limited current (TCLC) occurs for m > 2 with V^m^ dependence of current^38^.

The active layer low mobility causes inequality between the rate of collection of carriers at electrodes and rate of injection of carriers at electrodes which result SCLC to occur^39^ and this happens to be more dominant at the junctions.

To investigate and understand different charge transport mechanisms in our fabricated devices –Ag/F8-CdSe/P3HT/ITO and Ag/F8/P3HT/ITO– the power law is applied on Fig. 5 to obtain the log(*I)* vs log(*V)* graphs as shown in Fig. 8. Figure 8 shows curves with three distinct regions having different slopes. Each region (slope) represents a unique charge transport mechanism in the devices. In region-I, the slopes (m) of the curves for both the devices are 2.3 which could be rounded to 2, i.e., 2.3 ≈2 that indicates the SCLC or formation of the depletion region across the junctions. This is the region and charge transport scheme which is responsible for the rectification in the forward bias current and, therefore, the dominant conduction mechanism in the devices. In region-II, the slopes for Ag/F8-CdSe/P3HT/ITO and Ag/F8/P3HT/ITO are observed to be 3.8 and 1.26 ≈1, respectively, where the former represents the TCLC mechanism while the later express the ohmic conduction. Moreover, region-III has as slope of 1.43 for Ag/F8-CdSe/P3HT/ITO while 4.4 for Ag/F8/P3HT/ITO device, which points towards the ohmic and TCLC conduction mechanisms, respectively. The occurring of TCLC and ohmic regions are observed at different locations in the graphs obtained for Ag/F8-CdSe/P3HT/ITO and Ag/F8/P3HT/ITO devices which can be attributed to different levels of traps and their distribution in F8-CdSe QDs nanocomposite and F8. From Fig. 8, it can be seen that Ag/F8-CdSe/P3HT/ITO demonstrate TCLC mechanism in region-II which implies the traps present in F8-CdSe QDs nanocomposites are shallow traps (lower energy traps) and begin to fill earlier as compared to F8. Moreover, F8 exhibits ohmic conduction mechanism in region-II that indicates the presence of deep traps (higher energy traps), therefore, the injected charge carriers are not sufficient to fill these traps and hop between the deep energy traps; consequently, the current is proportional to the applied voltage. The current density in these regions is given by Eq. (6);

6 $${J}_{SCLC}=\frac{9{\varepsilon }_{s}{\mu }_{SCLC}{V}^{2}}{8{d}^{3}}$$

Here “µ_SCLC_” is charge carrier mobility in SCLC region, “ε_s_ = ε_0_ε_r_ is the dielectric constant of organic film where ε_0_ = 8.85 × 10^−14^ Fcm^−1^ and ε_r_ = 4–8 are the free space permittivity and organic film relative permittivity, respectively^40^, and *d* = 80 nm is the film thickness. The region where slope is greater than 2, the dominant mechanism is the trap-charge-limited current (TCLC) with exponential distribution of traps. So current density for traps is given by:

7 $${J}_{TCLC}=\frac{9{\varepsilon }_{0}{\varepsilon }_{r}\theta {\mu }_{TCLC}{V}^{2}}{8{d}^{3}}$$

Here “θ” is trapping factor and µ_TCLC_ is the charge mobility at TCLC region. Hence, the calculated values of µ_SCLC_ and µ_TCLC_ from Eqs. (6) and (7) are 2 × 10^−4^ cm^2^V^−1^s^−1^ and 1 × 10^−4^ cm^2^V^−1^s^−1^, respectively, for Ag/F8/P3HT/ITO device. Whereas, the measured values of µ_SCLC_ and µ_TCLC_ are 4 × 10^−4^ cm^2^V^−1^s^−1^ and 5 × 10^−4^ cm^2^V^−1^s^−1^, respectively, for Ag/F8-CdSe/P3HT/ITO SBD.

To study and understand the charge carriers’ conduction mechanisms in the device, there may be different kinds of phenomena involved. However, generally, there are two main conduction mechanisms; i. Electrode limited conduction mechanisms ii. Bulk-limited conduction mechanisms^41^.

Some of the conduction mechanisms depend on the electrical properties at the electrode/semiconductor or insulator junction. These are called electrode-limited or injection-limited conduction mechanisms. Whereas, the conduction mechanisms which are influenced by properties of the semiconductor material itself; are known as transport-limited or bulk-limited conduction mechanisms^42^.

Electrode limited conduction mechanism could be due to one of these processes which are Richardson-Schottky (RS) emission, Fowler-Nordheim tunneling, direct tunneling and thermionic-field emission. While, bulk limited conduction mechanism could be due to of one the mechanisms which are; Poole-Frenkel (PF) emission, hopping conduction, ohmic conduction, SCLC, ionic conduction and grain-boundary-limited conduction^43^.

Herein, the device structure can be assumed analogous to metal-insulator-metal (MIM) structure i.e., Ag/F8/P3HT/ITO. Where the Ag top contact and ITO bottom contact/substrate are highly conductive materials, P3HT is buffer layer/hole transport layer and F8 which is an ploymeric semiconductor which acts as an insulator (due to lower charge mobility as compared to inorganic semiconductors) to resemble MIM structure. Usually, the dominant bulk transport mechanism in organic semiconductors is due to hopping and/or SCLC which is studied and confirmed by analyzing Fig. 8. Hence, in the bulk limited conduction mechanisms, SCLC is found to be dominant conduction process in Ag/F8-CdSe/P3HT/ITO and Ag/F8/P3HT/ITO devices.

**Figure 9:**
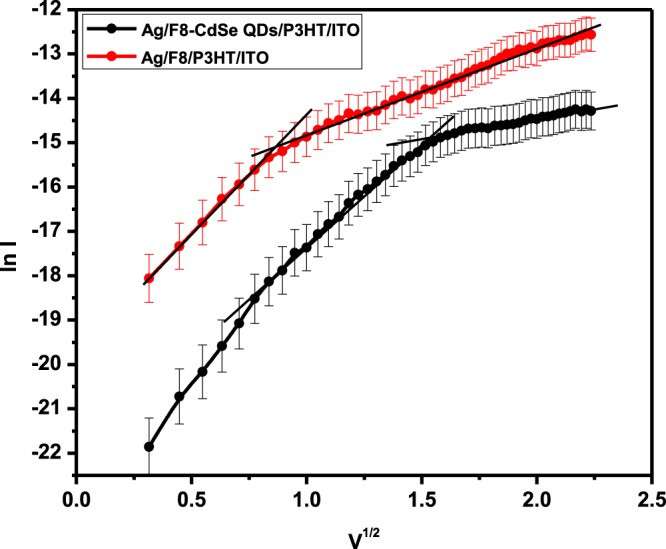
ln(*I)*-*V*^*1/2*^ characteristics of Ag/F8-CdSe/P3HT/ITO and Ag/F8/P3HT/ITO SBDs.

Figure 9 presents a relation between ln(*I)* against *V*^*½*^ is plotted in reverse bias for the Ag/F8/P3HT/ITO and Ag/F8-CdSe/P3HT/ITO SBDs respectively. The figure reveals that current in the junction is characterized by two linear segments via different slopes which might be interpreted in terms of the two field-lowering mechanisms, which are the RS and PF mechanisms. The RS mechanism is considered as barrier and/or electrode limited conduction whereas PF is a bulk limited process. Furthermore, RS mechanism is associated with the injection of charge carriers from the electrodes to the F8 and F8-CdSe layers by field assisted lowering of metal/F8 and metal/F8-CdSe potential barriers^44^. Whereas, in case PF process, charge carriers are released from the traps due to field assisted lowering of trap depths in the bulk of F8 and F8-CdSe films^45^. The current for both mechanisms is given by^46^;

For RS effect;

8 $${I}_{0}=A{A}^{\ast }{T}^{2}\exp \left(-\frac{{\phi }_{b}}{kT}\right)\exp \left(\frac{{\beta }_{RS}{V}^{1/2}}{kT{d}^{1/2}}\right)$$

and for PF effect;

9 $$I={I}_{0}\exp \left(\frac{{\beta }_{PF}{V}^{1/2}}{kT{d}^{1/2}}\right)$$

where *β*_*RS*_ is RS field lowering coefficient and *β*_*PF*_ is PF field lowering coefficient. For *β*_*RS*_ and *β*_*PF*_, the theoretical values can be calculated from the following expression:

10 $$2{\beta }_{RS}={\beta }_{PF}={\left(\frac{{q}^{3}}{\pi {\varepsilon }_{\circ }{\varepsilon }_{r}}\right)}^{1/2}$$

The relative permittivity (ε_r_) for F8 is 4 and the values of *β*_*RS*_ and *β*_*PF*_ are theoretically calculated from Eqs. (8) and (9) which are 3.8×10^−5^ eVm^1/2^ V^−1/2^ and 7.6×10^−5^ eVm^1/2^V^−1/2^, respectively.

From Fig. 9, for Ag/F8/P3HT/ITO device, the experimental values of *β*_*RS*_ and *β*_*PF*_ extracted from the slope of the curve at lower voltage region are 3.55×10^−5^ eVm^1/2^V^−1/2^ and 7.11×10^−5^ eVm^1/2^V^−1/2^, respectively, while at higher voltage regime their values are 1.28×10^−5^ eVm^1/2^V^−1/2^ and 2.55×10^−5^ eVm^1/2^V^−1/2^, respectively. Similarly, the experimental values of *β*_*RS*_ and *β*_*PF*_ are also calculated for Ag/F8-CdSe/P3HT/ITO device by finding the slopes of its graph at lower and higher voltage regions as shown in Fig. 9. Table 3 presents the theoretical and experimental values of *β*_*RS*_ as well as *β*_*PF*_ for Ag/F8/P3HT/ITO and Ag/F8-CdSe/P3HT/ITO devices. Hence, comparing the theoretical values of *β*_*RS*_ and *β*_*PF*_ with their corresponding experimental values at lower and higher voltage regions, it is found that the theoretical value of *β*_*Rs*_ is much closer to the experimental value of *β*_*Rs*_ at low voltage region for both the devices. The difference in the theoretical and experimental values of *β*_*Rs*_ for Ag/F8/P3HT/ITO device is 0.25; whereas for the Ag/F8-CdSe/P3HT/ITO device it is found to be 0.71. Hence, the RS conduction mechanism is a dominant process in Ag/F8/P3HT/ITO and Ag/F8-CdSe/P3HT/ITO devices. It means that the contribution of charge carriers injection from the metal electrodes to the organic layer is higher than the carriers released from the traps due to field assisted lowering of traps depths.

**Table 3:** Comparison of theoretical and experimental values of β.

S. No	Devices	Theoretical values		Experimental values			
		*β*_*RS*_ (eVm^1/2^ V^−1/2^)	*β*_*PF*_ (eVm^1/2^ V^−1/2^)	Lower voltage region		Higher voltage region	
				*β*_*RS*_ (eVm^1/2^ V^−1/2^)	*β*_*PF*_ (eVm^1/2^ V^−1/2^)	*β*_*RS*_ (eVm^1/2^ V^−1/2^)	*β*_*PF*_ (eVm^1/2^ V^−1/2^)
1	Ag/F8/P3HT/ITO	3.8 × 10^−5^	7.6 × 10^−5^	3.55 × 10^−5^	7.11 × 10^−5^	1.28 × 10^−5^	2.55 × 10^−5^
2	Ag/F8-CdSe/P3HT/ITO			3.09 × 10^−5^	6.01 × 10^−5^	0.58 × 10^−5^	1.16 × 10^−5^

The value of *ϕ*_*b*_ and R_s_ can also be found through Cheung and Cheung method^47^.

The Cheung’s functions are given as:

11 $$\frac{dV}{d\,\mathrm{ln}\,I}=n\frac{kT}{q}+I{R}_{s}$$

12 $$H(I)=I{R}_{s}+n{\phi }_{b}$$

where

13 $$H(I)=V-n\frac{kT}{q}\,\mathrm{ln}\left(\frac{{I}_{0}}{A{A}^{\ast }{T}^{2}}\right)$$

**Figure 10:**
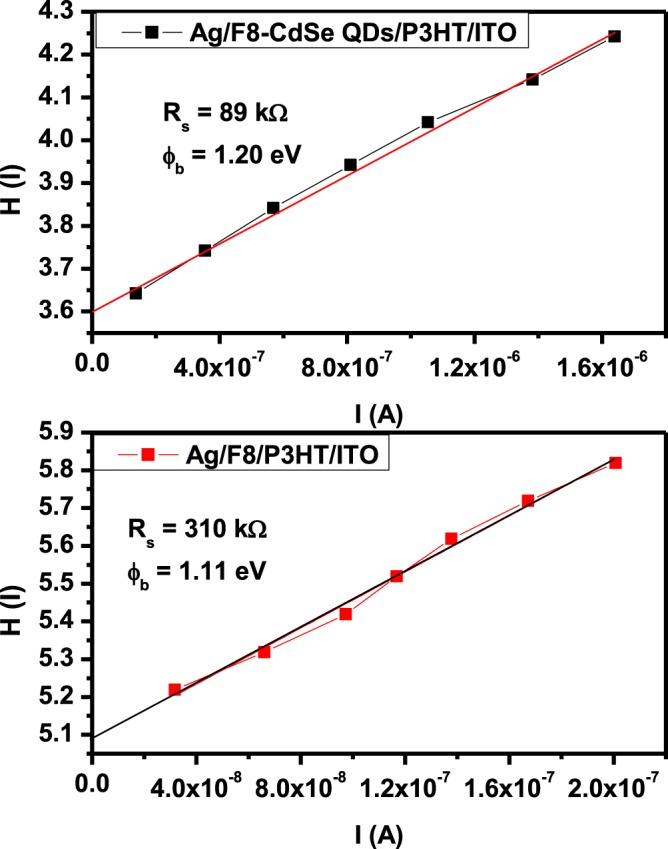
H (*I*) vs. *I* graph of Ag/F8-CdSe/P3HT/ITO and Ag/F8/P3HT/ITO Schottky Junction.

Figure 10 shows H (*I*) versus *I* plot and H(I) is given by Eq. (12). The values of junction parameters ϕ_b_ and R_s_ are extracted from the H (*I*) versus *I* graph and having the values 1.11 ± 0.05 eV and 310 ± 0.10 kΩ and 1.20 ± 0.05 eV and 89 ± 0.10 kΩ for Ag/F8/P3HT/ITO and Ag/F8-CdSe/P3HT/ITO device, respectively.

The values of junction parameters ϕ_b_ and R_s_ can also be determined by Norde’s method as Eq. (14) shows the relation for the Norde’s function:

14 $$F(V)=\frac{V}{\gamma }-\frac{kT}{q}\,\mathrm{ln}\left(\frac{I}{A{A}^{\ast }{T}^{2}}\right)$$

15 $${\phi }_{b}=F({V}_{0})+\frac{{V}_{0}}{\gamma }-\frac{kT}{q}$$

and

16 $${R}_{s}=\frac{kT(\gamma -n)}{q{I}_{0}}$$

**Figure 11:**
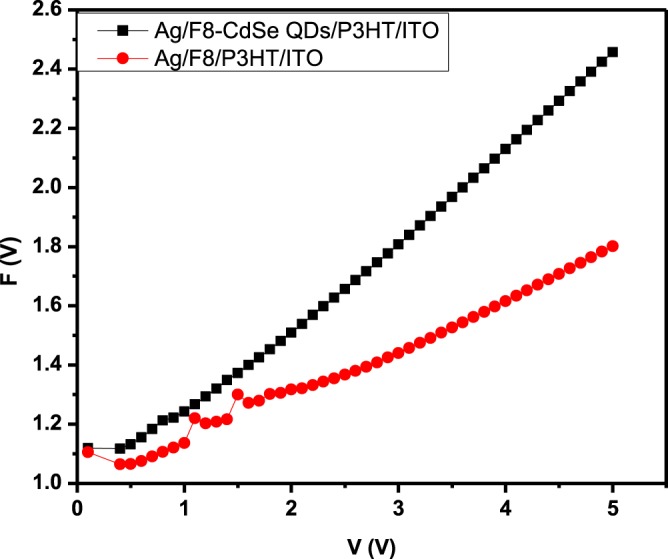
*F(V)-V* characteristics of Ag/F8-CdSe/P3HT/ITO and Ag/F8/P3HT/ITO SBDs.

Here *V*_0_ is the consistent value of voltage for which *F*(*V*_0_) has the lowest value of *F*(*V*), and at *V*=*V*_0_ the corresponding current is *I*_0_ and γ is an integral dimensionless quantity having value greater than that of ideality factor. The Norde’s function graph is shown in Fig. 11. The values of *ϕ*_*b*_ extracted from the graph were found to be 1.22 ± 0.05 eV and 1.10 ± 0.05 eV for Ag/F8-CdSe/P3HT/ITO and Ag/F8/P3HT/ITO junction devices, respectively. Whereas, the values *R*_*s*_ measured, for the former and later devices, were 90 ± 0.10 kΩ and 301 ± 0.10 kΩ, respectively. Table 4 describes the comparison of parameters for both of the devices i.e., Ag/F8-CdSe/P3HT/ITO and Ag/F8/P3HT/ITO, extracted from different characterization methods.

**Table 4:** Overall comparison of the parameters extracted from different methods.

Characterization Method	Device	(*n)*	*R*_*s*_ *(kΩ)*	*Φ*_*B*_ *(eV)*
**Conventional I–V**	Ag/F8/P3HT/ITO	4.03	269	1.03
	Ag/F8-CdSe/P3HT/ITO	2.38	78.2	1.17
**Cheung Function**	Ag/F8/P3HT/ITO	4.26	310	1.11
	Ag/F8-CdSe/P3HT/ITO	2.42	89	1.20
**Norde’s Function**	Ag/F8/P3HT/ITO	—	301	1.10
	Ag/F8-CdSe/P3HT/ITO	—	90	1.22

## Conclusions

The work carried out in this paper demonstrated enhancement in the microelectronic properties a SBD Ag/F8-CdSe QDs/P3HT/ITO made from the polymer matrix F8 blended with CdSe QDs. Two devices –Ag/F8/P3HT/ITO and Ag/F8-CdSe QDs/P3HT/ITO– were fabricated and characterized at the same ambient conditions. Both of the devices exhibited asymmetrical and rectifying *I–V* curves which confirm the formation of SBDs and depletion region across the junction. By comparing different microelectronic parameters extracted from conventional *I–V* method, we observed RR of 7.42 at ±3.5 V, ideality factor ~4.03, ϕ_b_ ~1.09 eV, R_s_ ~269 kΩ and *I*_0_ ~6.23 × 10^−10^ A for Ag/F8/P3HT/ITO SBD while RR of 142 at ±3.5 V, ideality factor ~2.38, ϕ_b_ ~1.17 eV, R_s_ ~78.2 kΩ and *I*_0_ ~2.39 × 10^−11^ A were measured for Ag/F8-CdSe QDs/P3HT/ITO device. To validate these extracted parameters of the SBDs, some of the parameters are measured by using Cheung functions and Norde’s method which are found in good agreement. The microelectronic parameters obtained from Ag/F8-CdSe QDs/P3HT/ITO SBD confirm the potential role of CdSe QDs in enhancing the device properties due to large surface to volume ratio and 3-dimensional quantum confinement effects of CdSe QDs. Improvement in charge carrier mobility is also observed in F8-CdSe QDs based SBD as compared to that of F8-alone based device. Optical bandgap was measured to be 1.88 ± 0.05 eV and photoluminescence (PL) spectrum showed broad band with a maximum peak at 556 nm for CdSe QDs. Hence, this study suggests CdSe QDs for its potential use in high performance SBDs, electronic and optoelectronic devices.
